# Fat-Containing Renal Cell Carcinoma Mimicking Angiomyolipoma: A Radiological and Histopathological Diagnostic Challenge

**DOI:** 10.7759/cureus.6721

**Published:** 2020-01-21

**Authors:** Amman Yousaf, Usman Nabi, Mohamed Lameir Hussein, Akram Twair, Mohamed ben Gashir

**Affiliations:** 1 Radiology, Hamad General Hospital, Doha, QAT; 2 Radiology, Hamad Medical Corporation, Doha, QAT; 3 Clinical Imaging, Hamad Medical Corporation, Doha, QAT; 4 Laboratory Medicine and Pathology, Hamad Medical Corporation, Doha, QAT

**Keywords:** renal cell carcinoma, angiomyolipoma, fat-containing renal tumors, calcifications, surgical excision, malignant renal tumors, benign renal tumors, case report, imaging, histopathology

## Abstract

A well-marginated fat-containing renal lesion gives strong suspicion of renal angiomyolipoma (RAML) that usually requires no surgical intervention. Radiologically, renal masses with a large amount of fat can rule out renal cell carcinoma (RCC). Calcifications are very infrequent in RAML. However, the presence of calcifications in a fat-containing renal lesion is highly suggestive of RCC. These lesions should undergo surgical resection and histopathological assessment to reach the right diagnosis and avoid poor outcomes if diagnosed late. We present a case of bilateral renal tumors, in which one of them radiologically contained abundant fat with calcifications on CT scan, which was confirmed to be an RCC on histopathological examination.

## Introduction

Abdominal imaging has been used extensively over the past decade in patients presenting with gastrointestinal and urological symptoms. With the increasing use of radiological modalities, there is a rising trend of diagnosis of solid renal tumors incidentally [1]. In a well-demarcated renal mass, the demonstration of fat on CT scans is highly suggestive of renal angiomyolipoma (RAML) [2]. However, renal masses with macroscopic fat can be malignant. Small renal cell carcinoma (RCC) with osseous metaplasia, large RCC with perirenal fat invasion, and Wilms tumor are the examples of malignant renal tumors that can contain fat [3]. The presence of calcifications in renal masses raises the suspicion of malignancy and is proven to be RCC in 40% of the cases, regardless of the pattern and number. Therefore, the presence of fat and calcifications are more consistent with RCC [4]. We are presenting a case with bilateral well-demarcated solid renal masses, one of which contained abundant fat and calcifications.

## Case presentation

Clinical history

A 69-year-old male with a history of hypertension presented to the emergency department with complaints of urinary frequency, urgency, and hesitation to pass urine. Physical examination was unremarkable. Laboratory tests showed high leukocytes in urine (19 WBC/hpf) and elevated prostate-specific antigen (PSA) of 14 ng/mL.

Radiological features

**Figure 1:**
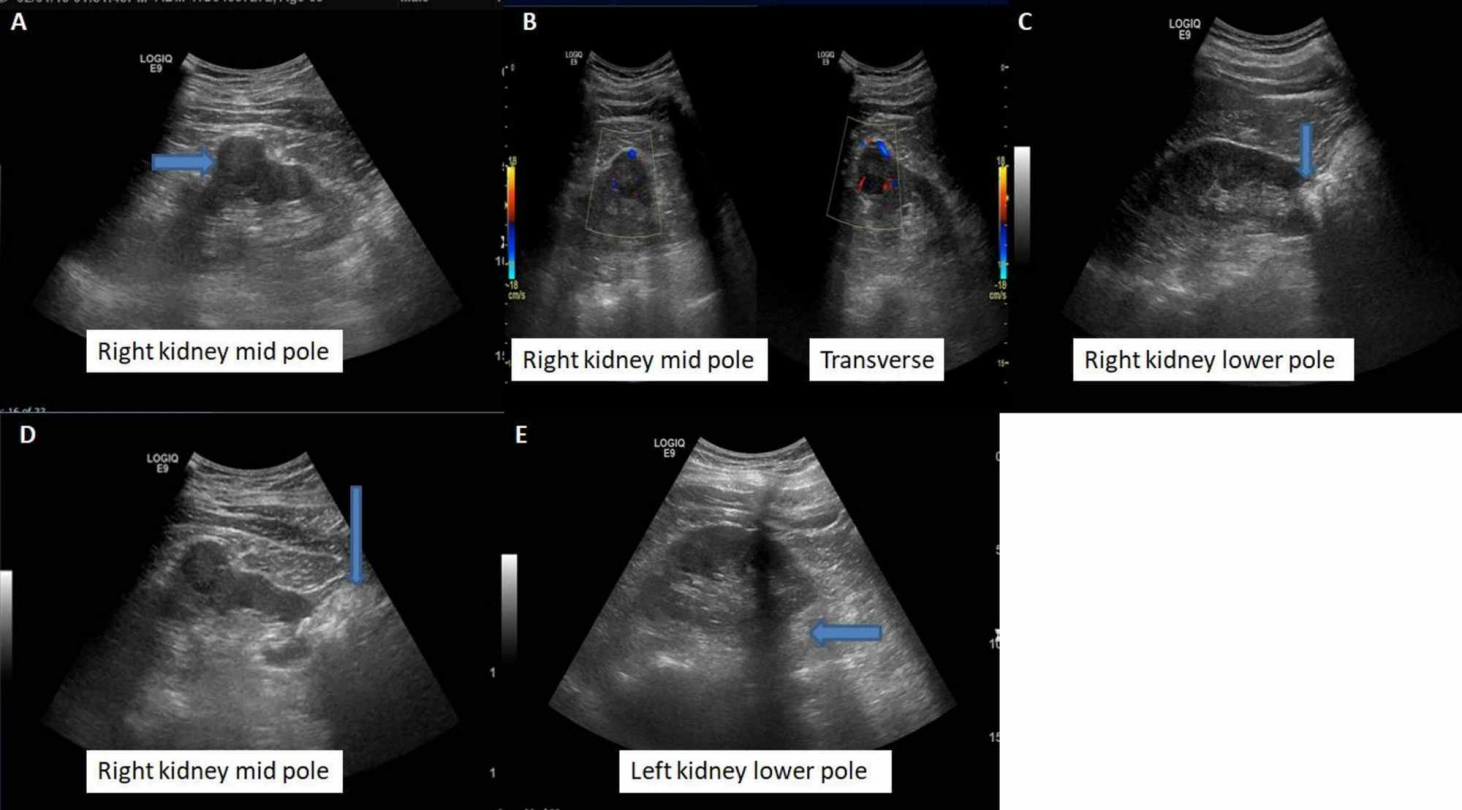
Ultrasound of the abdomen The right kidney demonstrates a hypoechoic mass lesion in its middle pole showing internal and peripheral vascularity measuring 25 x 22 mm (A and B). An ill-defined focal mass lesion can be seen in the right kidney lower pole with calcifications and posterior acoustic shadowing (C and D). A subtle hypoechoic round-shaped lesion can be seen at the medial aspect of the left kidney (E).

Ultrasound examination of the urinary tract showed an enlarged prostate with an estimated volume of 67 mL. In addition to this, two solid mass lesions were found incidentally in the right kidney. The first lesion was a well-defined hypoechoic mass lesion in the middle pole region, measuring 25 x 22 mm. A second hypoechoic lesion was identified in the lower pole measuring 10 cm and had calcifications as well. The left kidney also showed a small hypoechoic lesion in the lower pole (Figure 1).

The patient had undergone treatment for urinary tract infection and benign prostatic hypertrophy. On follow-up, repeated PSA showed a significant decrease in level to 6 ng/mL as compared with the previous reading of 14 ng/mL. Thus, the patient did not undergo prostatic biopsy.

**Figure 2:**
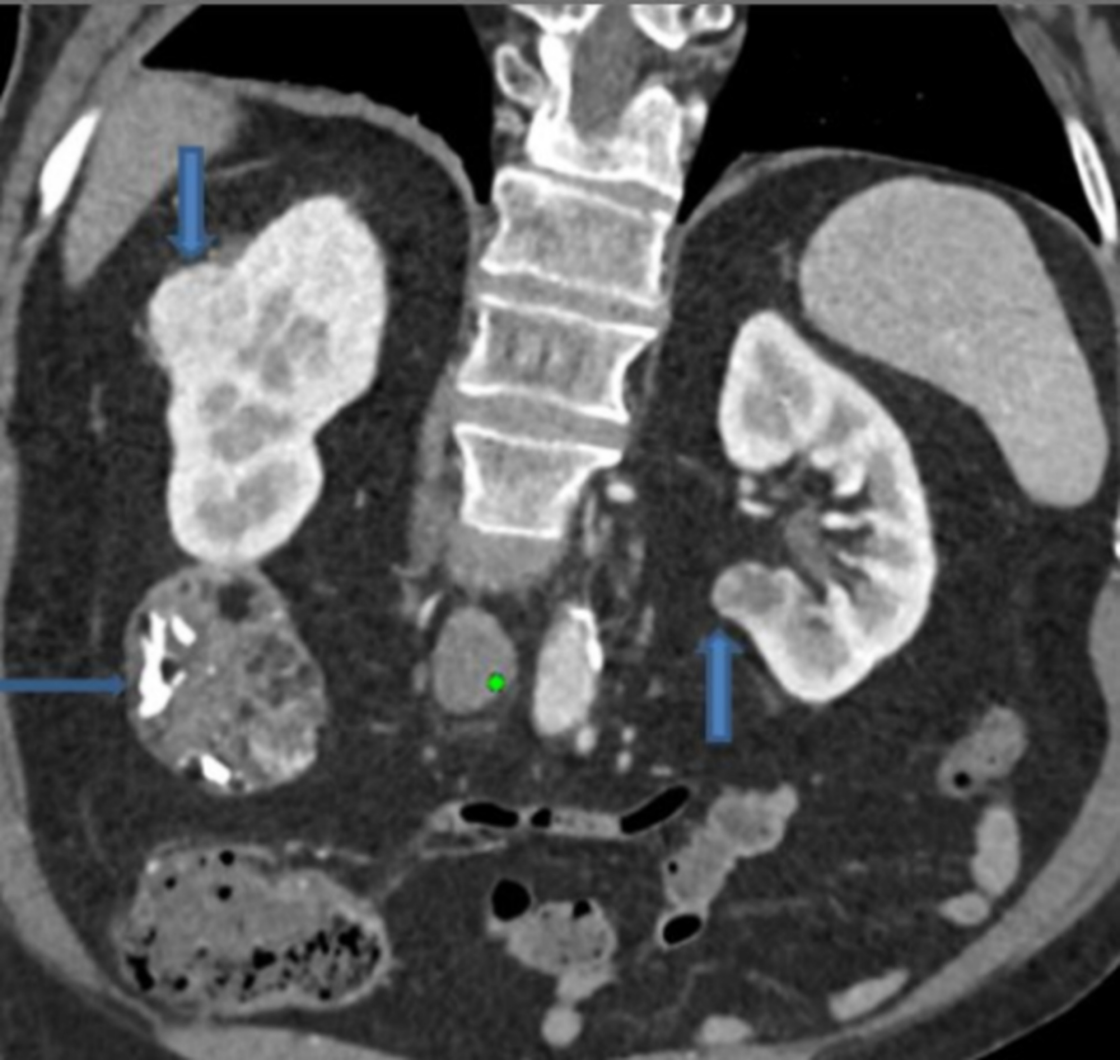
Contrast-enhanced CT scan of the abdomen and pelvis An oblique coronal view demonstrates all three lesions in the kidney. The one in the lower pole of the right kidney is the largest.

The patient underwent a contrast-enhanced CT scan for further characterization of the renal masses. CT scan confirmed three renal mass lesions, two in the right kidney and one in the left kidney (Figure 2).

**Figure 3:**
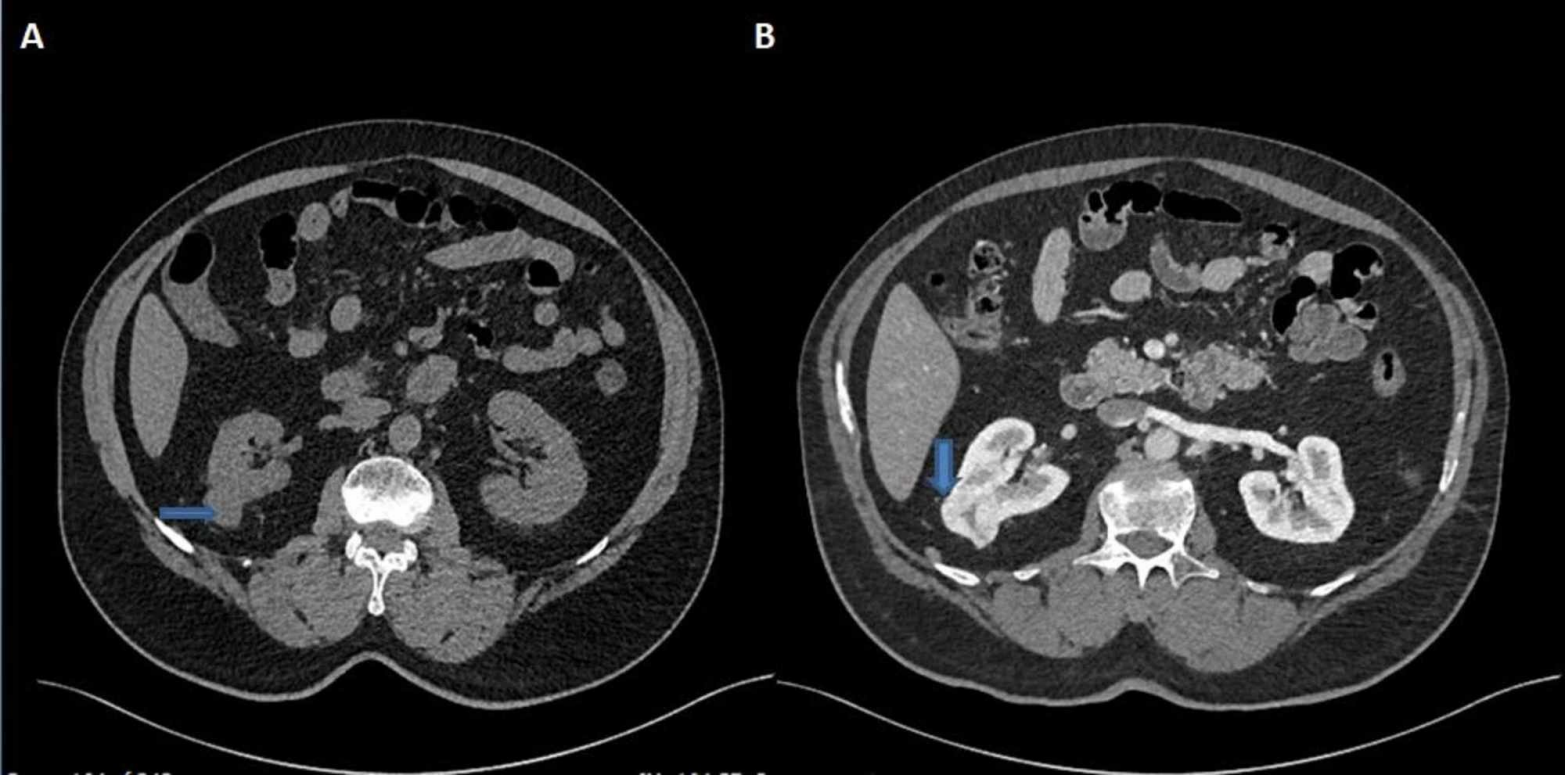
Axial sections of CT of the abdomen and pelvis It reveals an exophytic mass lesion measuring 27 mm in size in the interpolar region of the right kidney (A). It is demonstrating contrast enhancement with a central non-enhancing area (B).

There was an exophytic mass lesion in the middle pole of the right kidney, measuring 27 mm in diameter, and showed enhancement similar to the renal cortex on post-contrast images with a central non-enhancing area. This lesion showed contrast washout in the delayed phase (Figure 3).

**Figure 4:**
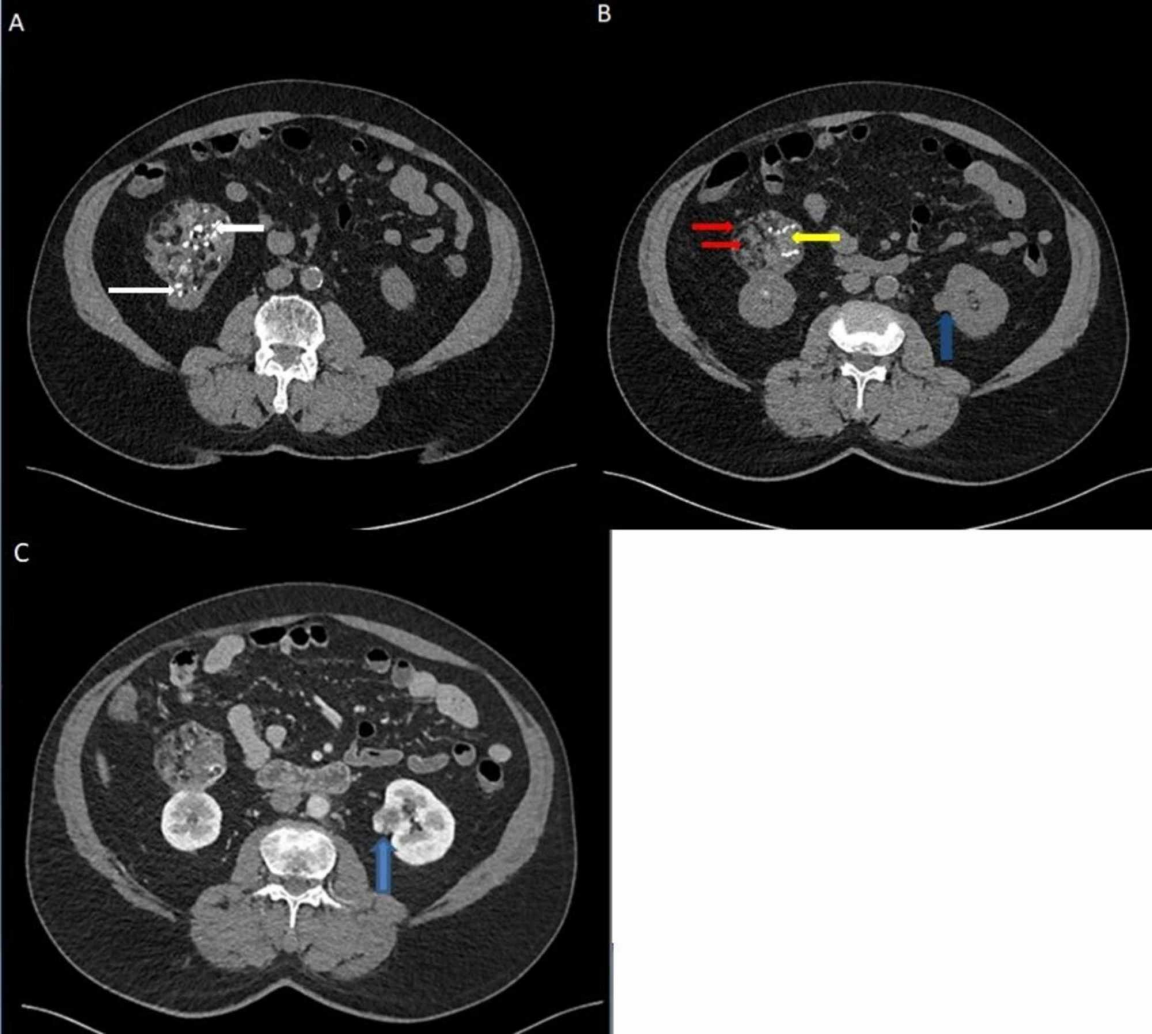
Axial sections of CT of the abdomen and pelvis at the level of lower poles of the kidneys A large exophytic fat-containing heterogeneous mass with focal calcifications (white arrows) in the lower pole of the right kidney, measuring 77 x 61 mm, can be seen (A-C). The hypodensities (red arrows) may explain the fatty contents, whereas the density of the other parts (yellow arrow) of the tumor is similar to the right kidney. A perceived heterogeneous faint contrast enhancement can be seen in the right kidney lesion (C). A suspicious small exophytic renal nodule measuring 14 mm in diameter is seen at the lower pole of the left kidney as well (blue arrow in 4B), showing heterogeneous enhancement (blue arrow in 4C).

The lesion in the lower pole of the right kidney was a large (77 x 61 mm) exophytic heterogeneous lesion with foci of macrocalcifications. It showed heterogeneous contrast enhancement and multiple areas of fatty components. In the lower pole of the left kidney, there was a small lesion measuring 14 mm in diameter, showing heterogeneous contrast enhancement with a central non-enhancing area (Figure 4).

The presence of an extensive amount of fat in the lower pole lesion of the right kidney suggested the diagnosis of RAML. However, the presence of calcifications, which is an infrequent observation in RAML, raised the possibility of a massive fat-containing RCC. These lesions did not invade the pelvicalyceal system or renal veins on either side. There were no sizeable para-aortic lymph nodes.

The multidisciplinary team agreed on the decision to perform bilateral partial nephrectomy in a step-wise manner. The patient underwent right partial nephrectomy by the uro-oncology team, which went uneventful.

Histopathological features

The histopathology was quite challenging. Specimens from the middle and lower poles of the right kidney showed a large amount of fat. They showed two different histological features with almost identical morphological and immunohistochemical features.

**Figure 5:**
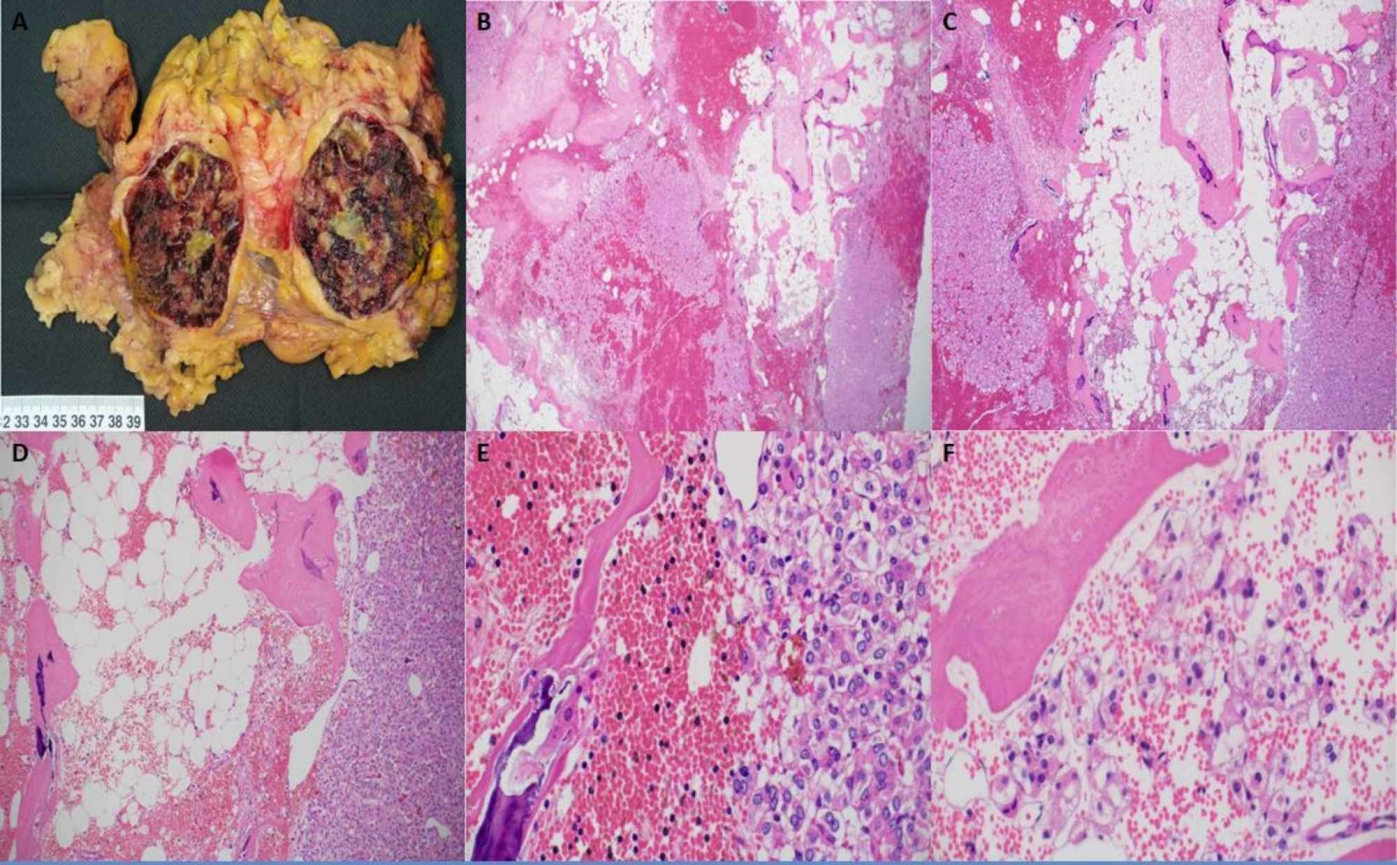
Histopathological features of the right kidney specimen (lower pole) A well-defined mass is occupying most of the specimen that has hemorrhagic areas alternating with tan whitish areas, yellow areas, and cystic spaces (A). Microscopic examination shows a heterogeneous tumor comprising areas of mature adipose tissue, hemorrhage, dystrophic calcifications with foci of ossification, hyalinized fibrous stroma, patchy sheets of pleomorphic epithelial cells with monomorphic, and nuclei. Some of the epithelial cells are present within the mature adipose tissue, and other epithelial islands are present within hemorrhagic areas (B-F).

Gross examination of the specimen from the lower pole of the right kidney (Figure 5A) revealed an unoriented mass surrounded by fatty tissue weighing 560 g and measuring 13 x 10 x 9 cm. Slicing revealed a well-defined mass occupying most of the specimen measuring 8 x 6.5 x 6.5 cm. It had a variegated cut surface showing hemorrhagic areas alternating with tan whitish areas, yellow areas, and cystic spaces. The periphery of the mass showed homogenous whitish regions that might represent compressed renal tissue. The tumor appeared to be abutting the surrounding fatty tissue but not infiltrating it. Microscopic examination (Figure 5B-5E) showed a heterogeneous tumor comprising areas of mature adipose tissue, hemorrhage, dystrophic calcifications with foci of ossification, hyalinized fibrous stroma, patchy sheets of pleomorphic epithelial cells with monomorphic nuclei, cholesterol clefts, and bone marrow elements with trilineage hematopoiesis. The epithelial cells had pleomorphic round nuclei with mild-to-moderate atypia and conspicuous nucleoli (grade 3). Some of the epithelial cells were present within the mature adipose tissue, and other epithelial islands were present within hemorrhagic areas. There was a thin rim of residual fibrotic renal parenchyma in the periphery of the tumor adjacent to adipose tissue. Immunohistochemistry showed that the epithelial cells are positive with Ae1/Ae3, patchy MNF116, PAX8, and RCC, but negative with CK7, S100, HMB45, MelanA, PAX2, SMA, desmin, vimentin, CAIX, CD117, synaptophysin, CD56, chromogranin A, CD10, TFE3, inhibin, and E-cadherin (Figure 5).

**Figure 6:**
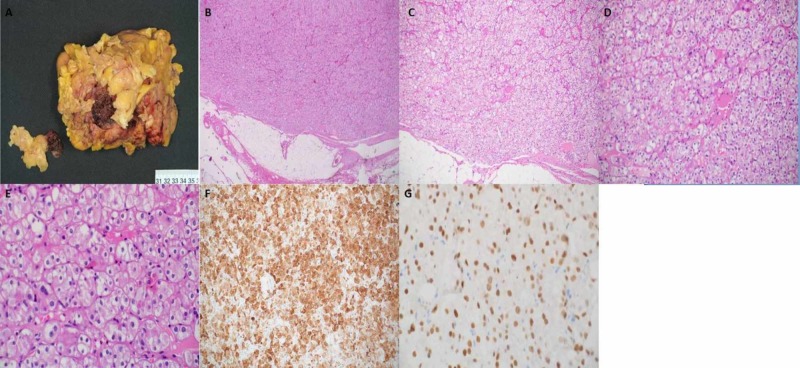
Histopathological features of the right kidney specimen (middle pole) An irregular piece of fibrofatty tumor that contains a hemorrhagic mass can be seen (A). Microscopy shows a homogeneous tumor comprising sheets and nests of epithelial cells with clear eosinophilic cytoplasm, round nuclei with mild-to-moderate atypia and conspicuous nucleoli (B-E). Immunohistochemistry shows that the tumor cells are positive with CD10 (F) and PAX8 (G).

Gross examination of the specimen from the middle pole of the right kidney (Figure 6A) revealed an irregular piece of fibrofatty tissue measuring 8 x 7 x 2.8 cm containing a hemorrhagic mass measuring 3.5 x 3 x 2 cm. Microscopic examination (Figure 6B-6E) showed a homogenous tumor comprising sheets and nests of epithelial cells with clear eosinophilic cytoplasm, rounded nuclei with mild-to-moderate atypia, and conspicuous nucleoli. Immunohistochemistry (Figure 6F, 6G) showed that the tumor cells were positive with Ae1/Ae3, MNF116, CD10 (Figure 6F), E-cadherin, patchy CK7, patchy PAX8 (Figure 6G), and patchy CD56, but negative with vimentin, HMB45, S100, MelanA, PAX2, CD117, CAIX, chromogranin A, TFE3, RCC, CK20, and inhibin (Figure 6).

Based on these features, the differential diagnoses included multifocal RCC (unclassified), multifocal clear cell RCC (eosinophilic variant), multifocal chromophobe RCC, and oncocytoma. Another possibility was RCC infiltrating angiomyolipoma, but the morphology and immunohistochemical features were against RAML. Based on morphological and immunohistochemical features, the final diagnosis in both specimens was RCC.

Follow-up

Due to the malignant features of the tumors on histopathology, the patient underwent a CT scan of the thorax, abdomen, and pelvis six months after the surgery to look for recurrence. Fortunately, the CT scan showed no recurrence or residual tumor on the right side. Although the left-sided tumor was of the same size in the repeated CT scan, the uro-oncology team offered the patient biopsy or partial nephrectomy. But, the patient refused to take any further management.

## Discussion

RAML, fat-containing RCC, lipoma, liposarcoma, and oncocytoma are the renal tumors that contain fat [2]. Our primary focus in this case report is RCC and RAML. RCC is the most common malignant solid renal mass. It represents more than 90% of renal malignancies and 2-3% of all cancers. Its peak age of presentation is 60 to 70 years [1]. The classic triad of clinical presentation is a palpable mass, hematuria, and flank pain, which are not commonly present altogether until the late stage of the disease [1]. In children, there is no gender predominance, whereas in adults, RCC is more predominant in males [5]. Tobacco smoking, hypertension, dialysis-related cystic disease, and obesity are the most common risk factors of RCC [1]. RCC is also associated with rare hereditary syndromes, i.e., Von Hippel-Lindau syndrome and hereditary papillary RCC. Prognosis depends on the TNM stage, grade, and RCC subtype [6].

Incidental diagnosis of renal tumors is becoming more common due to recent advancements in radiological investigations and increasing trend in the use of the diagnostic techniques for common diseases. Incidentally diagnosed cases have a better prognosis due to the early stage of detection and low grade [7]. A well-demarcated hyperechoic renal lesion on ultrasound is highly suggestive of an angiomyolipoma. However, this is not specific and therefore demands further investigation with a contrast-enhanced CT of the abdomen and pelvis. The presence of a well-marginated renal mass containing a large amount of fat makes the diagnosis of RAML and needs no surgical intervention [8].

One of the following mechanisms can explain the reason behind the presence of fat in RCC. Lipid producing necrosis, osseous metaplasia, and entrapment of sinus fat by an extensive irregular RCC can explain the mechanism of the existence of fat within RCC [9]. RAML with gross fat can have variable areas of hemorrhage, necrosis, cystic changes, and, rarely, calcifications [10]. The presence of calcifications within a renal lesion raises the suspicion of RCC. The calcifications are present in 30% of cases of RCC [11]. For such renal tumors containing fat with calcifications, further management by partial or radical nephrectomy and confirmation of diagnosis by histopathology is the mainstream of treatment [3].

## Conclusions

The confirmation of a hyperechoic (fatty) renal mass with a CT scan is a crucial step in further evaluation as it can prevent surgery. The presence of calcifications in fat-containing renal mass is highly suggestive of RCC. The management of these lesions is by resection and histopathological confirmation. Also, fat-containing tumors involving perirenal or renal sinus fat, large irregular mass containing areas of small fat necrosis, lymph node involvement, or venous invasion raise the suspicion of RCC.
